# Dietary Leucine Supplementation Improves Muscle Fiber Growth and Development by Activating AMPK/Sirt1 Pathway in Blunt Snout Bream (*Megalobrama amblycephala*)

**DOI:** 10.1155/2022/7285851

**Published:** 2022-12-21

**Authors:** Mang-mang Wang, Hui-xing Guo, Yang-yang Huang, Wen-bin Liu, Xi Wang, Kang Xiao, Wei Xiong, Hao-kun Hua, Xiang-fei Li, Guang-zhen Jiang

**Affiliations:** Key Laboratory of Aquatic Nutrition and Feed Science of Jiangsu Province, College of Animal Science and Technology, Nanjing Agricultural University, No. 1 Weigang Road, Nanjing 210095, China

## Abstract

This research is aimed at evaluating the effects of leucine supplementation on muscle fibers growth and development of blunt snout bream through a feeding trial and a primary muscle cells treatment. An 8-week trial with diets containing 1.61% leucine (LL) or 2.15% leucine (HL) was conducted in blunt snout bream (mean initial weight = 56.56 ± 0.83 g). Results demonstrated that the specific gain rate and the condition factor of fish in the HL group were the highest. The essential amino acids content of fish fed HL diets was significantly higher than that fed LL diets. The texture (hardness, springiness, resilience, and chewiness), the small-sized fiber ratio, fibers density, and sarcomere lengths in fish all obtained the highest in the HL group. Additionally, the proteins expression related with the activation of the AMPK pathway (p-Ampk, Ampk, p-Ampk/Ampk, and Sirt1) and the expression of genes (myogenin (*myog*), myogenic regulatory factor 4 (*mrf4*) and myoblast determination protein (*myod*), and protein (Pax7) related to muscle fiber formation were significantly upregulated with increasing level of dietary leucine. *In vitro*, the muscle cells were treated with 0, 40 and 160 mg/L leucine for 24 h. The results showed that treated with 40 mg/L leucine significantly raised the protein expressions of BCKDHA, Ampk, p-Ampk, p-Ampk/Ampk, Sirt1, and Pax7 and the gene expressions of *myog*, *mrf4*, and myogenic factor 5 (*myf5*) in muscle cells. In summary, leucine supplementation promoted muscle fibers growth and development, which may be related to the activation of BCKDH and AMPK.

## 1. Introduction

The muscle is the structural tissue and motor organ of fish, and an important protein source of human food [1, 2]. From the perspective of developmental molecular biology, aquaculture with fish as an important object is essentially based on the actual ecological conditions, utilizing appropriate method to regulate the hyperplasia and hypertrophy of fish muscle fibers, thereby promoting the growth and development of muscle tissue, optimizing muscle quality, reaching the purpose of improving the efficiency of production, and satisfying market demand [3, 4]. Moreover, the process of differentiation, assembly, and regulation of muscle fibers is essentially the result of the expression of related functional genes and the interaction of regulatory factors in the process of fish growth and development [5]. Therefore, studying the internal relationship between the differentiation of fish muscle fibers and the temporal and spatial expression of related functional genes, as well as the action mechanism of related regulatory factors, has potential application guiding significance for promoting the rapid growth of cultured fish and improving the efficiency of aquaculture [6–9]. Nowadays, nutrients are considered to be one of the most important factors affecting meat quality traits [10, 11], but the research on the relationship between nutrients and flesh quality is not sufficient [12, 13]. Researching the mechanisms of how nutrients cause changes in muscle fiber growth and development is also important for improving fish flesh quality [14].

Leucine is a functional amino acid, which plays an important role in the growth and health of animals [15]. It was reported that acute supply of leucine alone upregulated slow fiber gene expression in skeletal muscles in immature animals [16]. Haegens et al. [17] showed that increasing leucine concentration in the medium could promote myogenic protein synthesis and myosin heavy chain (MyHC) expression level in skeletal muscle cells. Similarly, it has been reported that lack of leucine could destroy the differentiation of mammal myoblasts and primary satellite cells, which was related to the changes of the expression patterns of the myogenic regulatory factors (MRFs) [18]. At present, the research explored the effect of leucine on the muscle fibers growth and development of fish were rare, which mostly focuses on growth, basic body composition, amino acid metabolism, and so on [19]. However, according to the above mammalian studies, it can be seen that leucine can affect the muscle fibers growth and development.

Blunt snout bream (*Megalobrama amblycephala*) is an important herbivorous freshwater fish from China with a small head and high meat content [20]. Favored for its great growth performance, good resistance to diseases and high dressout percentage [21, 22]. At present, some research provided evidences that the content of leucine with 1.61%-2.55% in the diet was beneficial for blunt snout bream, and the content of 1.61% leucine in the diet was recommended as the optimum according to quadratic regression analysis of specific growth rate (SGR) [23]. Nevertheless, the leucine requirement of blunt snout bream did not combine the effect of leucine on muscle formation and quality to determine although muscle tissue is the major edible part of fish and an important factor reflecting its economic benefits [2]. This effect was similarly omitted in studies analyzing leucine requirements in most fish [19, 24–26]. Accordingly, exploring how leucine influences muscle growth and development in *Megalobrama amblycephala* is helpful to improve fish quality and economic benefits.

In addition, the effect of leucine on fish follows the principle of dose effect [27–29]. Only when leucine is supplied in an appropriate range can it be effectively utilized by fish [30–32]. Therefore, the research combined *in vivo* and *in vitro* strategies to evaluate the effect of supplementary leucine within the recommended range on muscle texture, muscle growth and development related gene expression and protein expression, and to explored the pathways that may influence muscle formation in blunt snout bream.

## 2. Materials and Methods

The fish involved in this study were approved by the Animal Care and Use Committee of Nanjing Agricultural University (Nanjing, China) (permit number: SYXK (Su) 2011-0036). All experimental procedures involving animal care were kept conforming to the Guidance of the Care and Use of Laboratory Animals in China.

### 2.1. Diet Preparation

Two experimental diets, with fish meal and seed cake as protein sources, fish oil, and soybean oil as lipid sources, wheat bran and wheat middlings as carbohydrate sources, supplemented with vitamins and minerals, and added the corresponding concentrations of glycine to control the concentration of leucine (Table 1). In the previous studies of blunt snout bream, it can be seen that the SGR was highest in the group with 2.15% leucine in the diet, followed by the group with 1.61% leucine, and used the second-order polynomial regression model at 95% of maximum response to estimate the optimum dietary leucine requirement for juvenile blunt snout bream was 1.61% based on SGR [23]. Therefore, in this trial, 1.61 percent of leucine (LL) was used in the control diet, while the other diet had a leucine content of 2.15% (HL). The leucine used in this trial was crystalline l-leucine (Shandong Gukang Biological Engineering Co., Ltd., Shandong, China). All the raw ingredients were mixed evenly step by step to form a soft dough which was pelleted using a Pillet Mill (Guangyuan Engineering Co., Ltd., Shandong, China) with a 2 mm diameter. Then dried the diets at 28°C and sealed at 4°C for later use.

### 2.2. Experimental Procedure and Sample Collection

The test fish were supplied by the Fish Hatchery of Yangzhou (Jiangsu, China). Before the trial, all fish were fed a commercial diet (containing 32% protein and 5% lipid) in experimental conditions by hand-fed for 2 weeks. Following the 24 h starvation, 96 fish were randomly distributed into 8 floating net cages (12 fish per cage and 4 replicates for each diet), whose average body weight was 56.56 ± 0.83 g. The growth trial was carried out in the floating net cages (L: W: H, 1 × 1 × 1.5 m) in pond the same as Cao et al. [33]. Hand-fed the test fish thrice a day (7: 00, 12 : 00 and 17 : 00) [34], feed intake was noted. Recorded the number of dead fish and the weight of them. The water temperature (27-31°C), dissolved oxygen (5.0 mg/L) and pH (7.5-8.0) were recorded during the eight-week trial.

At the end of the eight-week trial, fasted the fish for 24 h, then recorded total fish weight and number in each cage. Captured three fish randomly from each cage and anesthetized them in MS-222 (100 mg/L, Sigma, USA). Measured the body length and body weight first. Then dissected the fish, respectively, collected and weighed viscera and liver. After removal of scales and skin, taken two cross sections of muscle (2-3 mm thick) from the dorsal fin of ipsilateral, one fixed in 4% paraformaldehyde solution and the remaining fixed in 2.5% glutaraldehyde, all stored at 4°C for subsequent analysis. In addition, taken 1 cm thick cubes from the dorsal side and measured texture analysis within 24 h. Dissected white muscle of dorsal from each fish and stored at −80°C until further analysis.

### 2.3. Amino Acid Profiles

Amino acid profiles in the muscle were calculated by high-performance liquid Chromatograph (Hitachi, Tokyo, Japan) following the procedures published by Lopez-Cervantes et al. [35]. Dietary leucine levels were determined as described above.

### 2.4. Texture Analysis

Texture measurements were conducted using the TA-XT Plus texture Analyzer from Stable Micro Systems, Surrey, England, which was controlled by a computer software (Stable Micro Systems Version 6.0, London, UK) to record data. According to Veland and Torrissen [36], the texture profile analysis (TPA) model was set to a pretest velocity of 5 mm/s, a test velocity of 1 mm/s, and a posttest velocity of 5 mm/s, with a deformation of 50% of the muscle thickness, interval between two compressions: 60s. Four samples were randomly selected from each group for measurement, and each sample was measured in parallel four times. After excluding the maximum and minimum values, the average value was taken. Texture indicators mainly include hardness, springiness, chewiness, cohesiveness, adhesiveness, gumminess, and resilience. For the definition of each parameter, please refer to the study of Nishinari et al. [37].

### 2.5. Texture Analysis of Muscle Samples

#### 2.5.1. Morphological Quantitative Analysis

The process of making paraffin sections refers to the research of Namei et al. [38]. The white muscle tissues after fixation in fixative (4% paraformaldehyde solution) for 24 h were dehydrated with 75%, 85%, 95%, and 100% absolute ethanol in sequence, then transparent with xylene, and immersed in wax for 2.5 h. The paraffin-impregnated tissues were embedded in a machine from Leica, Berlin, Germany, and after solidification, then a thickness of 6 *μ*m paraffin sections were made in a microtome (Leica RM2016, Berlin, Germany). After routine HE (hematoxylin and eosin) staining and neutral gum mounting, observed the histological structure of muscles with the microscope (Nikon Eclipse 80i, Tokyo, Japan), and captured the images with a specific camera (Nikon DS-U2, Tokyo, Japan). Three sections were selected for each group, and each section was subjected to four quantitative histological analyses by the software (Image-Pro Plus, media cybernetics, USA).

#### 2.5.2. Ultrastructural Analysis

White muscle samples soaked in 2.5% glutaraldehyde solution for more than 24 h were rinsed three times with PBS (phosphate buffer solution) and then with OsO4 (1% osmium tetroxide) fixed. After rinsing with double-distilled water, they were dehydrated with 50%, 70%, 80%, and 90% alcohol in sequence, and then embedded in the entrapment media (epoxy resin Epon812). Sections with the thickness of 70 nm were made with an ultramicrotome (EM-UC6, Leica, Germany) and counterstained with 3% uranyl acetate-lead citrate. Sarcomere length (SL) measurement with a software (Image-Pro Plus, media cybernetics, USA) after observation and photographing under transmission electron microscope (Hitachi, H-7500, Japan). Captured four images at different locations of each section, and read each picture four times.

### 2.6. *In Vitro* Treatment

#### 2.6.1. Primary Muscle Cell Culture

Select healthy *Megalobrama amblycephala* (~50 g body weight) from the aquatic base of Nanjing Agricultural University (Jiangsu, China). Soaked the fish in 70% alcohol for 1-2 min, then carefully obtained some the cubes of muscle (1.5-2 mm^3^) above the lateral line of the fish near the dorsal fin under sterile conditions. The muscle cubes were washed in sterile PBS (2-3 times), then placed in AIM (6% antibiotic incubation medium) prepared ahead of time. After two hours, inoculated the muscle cubes in 25cm^2^ plastic culture flasks (Corning, NY) and cultured in full growth media (M199 medium supplemented with 20% FBS) (refreshed every 1-2 days). The incubator temperature was 28°C and the CO_2_ concentration was 5%. The primary muscle cells grew to 80-90% confluence in about 16 days, added 2 mL 0.25% trypsin-EDTA (Gibco) to digest, and depended on experimental needs to subculture at 1 : 2 or 1 : 3. The process of primary muscle cell culture of blunt snout bream was based on Wang et al. [39] research.

#### 2.6.2. Primary Muscle Cell Pretest

**Figure 1:**
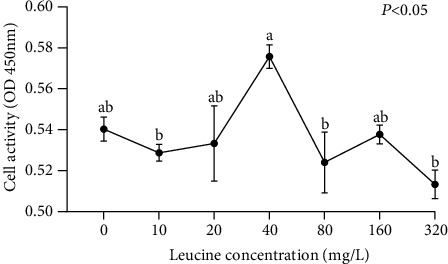
The activity of blunt snout bream muscle cells cultured in the seven medium of leucine levels. Values are represented as mean ± SEM (*n* = 4) by vertical bars. Mean values with different letters indicate significantly different (*P* < 0.05).

Muscle cells viability were evaluated by Cell Counting Kit-8 (CCK-8, APExBio, USA) in order to analyze the effect of different concentrations of leucine on the proliferation of muscle cells to confirm the optimal leucine concentration, and to carry out follow-up experiments (Figure 1). The leucine (S20044, Yuanye, China) was designed as 7 concentration gradients of 0 (the media), 10, 20, 40, 80, 160, 320 mg/L. This leucine was soluble in the medium used in this experiment. The cell suspension (100 *μ*L/well) was seeded in a 96-well plate, and after the cells adhered, the cell culture medium was replaced, and equal volumes of leucine with different concentrations were added for culture. After culturing for 24 h, 10 *μ*L of CCK-8 solution was added to each well, placed in a 37°C cell incubator for 3 h, and then taken out, and the absorbance value of each well at 450 nm was detected with a microplate reader, with 4 replicates in each group.

#### 2.6.3. Primary Muscle Cell Formal Treatment

The final concentrations of leucine were set as 40 mg/L (40 mg/L group, LM) and 160 mg/L (160 mg/L group, LH), and the media was control group (control group, LC). Inoculated muscle cells in six-well plates at the density of 10^6^ cells/well and added different media, every treatment had four replicates. At sample collection, muscle cells per well were rinsed with PBS three times. The obtained samples are processed appropriately to the activity of branched-chain *α*-ketoacid dehydrogenase complex (BCKDH) analysis, gene expression analysis, and western blot analysis. The activity of BCKDH was determined with an assay kit (GMS50931.1, GENMED, USA) according to the instructions.

### 2.7. Quantitative RT-PCR

Total RNA was extracted from the white muscles and cells following the protocols of Trizol reagent (Vazyme, Nanjing, China), and dissolved in DEPC-treated water. Then calculated quantity and purity of them according to the method of [40]. Following the manufacturer's instructions, cDNA was generated from 500 ng DNase-treated RNA by a qPCR Kit (Vazyme, Nanjing, China), then diluted with DEPC-treated Water and stored at -20°C until use. The endogenous reference gene was *ef1α* (elongation factor 1 alpha). Designed Gene primers from available sequences using Primer 5 software (Table 2).

Real-time qPCR assays were performed with a real-time detector (BIO-RAD, USA). The assays were performed with a reaction mix of 20 *μ*L per sample, each of which contained 2 *μ*L template (100 ng cDNA), 10 *μ*L 2 × AceQ Universal SYBR qPCR Master Mix (Vazyme, Nanjing, China), 0.4 *μ*L of each primer (10 *μ*mol L^−1^) and 7.2 *μ*L dH_2_O. The procedure was set as follows: initial denaturation at 95°C for 5 min followed by 40 cycles, annealing at 95°C for 10 s and a final extension at 60°C for 30 s, followed by a melt curve analysis of 15 s from 95 to 60°C, 1 min for 60°C, and then up to 95°C for 15 s. The expressions of target gene were calculated by using the 2^−*ΔΔ*CT^ method [41] with *ef1α* as the calibrator.

### 2.8. Western Blot

The operating procedures of western blot derived from Shi et al. [42]. The samples of muscle cells and white muscle were soaked, respectively, in RIPA lysis buffer (#ab156034, Abcam, UK), and grinded with a low temperature tissue grinder (JXFSTPRP-CL, China) to lyse to abstract total protein of them. Determine protein concentration with a BCA protein assay kit (Beyotime, China). Proteins in heat-denatured protein lysates (20 *μ*g of protein) were separated by reducing SDS-PAGE electrophoresis and then transferred onto the PVDF membrane. After blocking in 5% bovine serum buffer for 2 h, the bands were incubated with primary antibody overnight at 4°C. After secondary antibody incubation, visualized the target proteins using chemiluminescence reagent (Bioscience, China). Detected the bands of proteins with a luminescence image analyzer (Fujifilm LAS-3000, Japan) and captured images. The expressions of the target protein were quantified in a software (Image-J, National Institutes of Health, USA).

The antibodies used were as follows: the primary antibodies GAPDH (#ab8245, Abcam, UK), BCKDHA (DF13663, Affinity, China), p-Ampk (#ab131357, Abcam, UK), Ampk (#ab3759, Abcam, UK), Sirt1 (#ab189494, Abcam, UK), Pgc-1*α* (#AF5395, Affinity, China), and Pax7 (AF7584, Affinity, China). Goat Anti-Mouse IgG H&L (#ab6728, Abcam, UK) and Goat Anti-Rabbit IgG H&L (#ab6721, Abcam, UK) were the second antibodies.

### 2.9. Immunofluorescence (If)

The paraffin sections of muscle tissue were placed in citrate antigen retrieval solution (P0081, Beyotime, China) for antigen repair. Washed with PBS and blocked with bovine serum albumin (BSA) for 30 min. The primary antibody BCKDHA (DF13663, Affinity, China) and Pax7 (AF7584, Affinity, China) was incubated overnight at 4°C, washed with PBS the next day, and then incubated with the secondary antibody (S0006, Affinity, China) for 1 h at room temperature. After washing with PBS, DAPI (4,6-diamino-2-phenylindole) (C1005, Beyotime, China) was used to dye the nucleus, then observed under microscope (LSM900, ZEISS, Germany) and captured photographs on computer. For the detailed process, please refer to the research of Guo et al. [43].

### 2.10. Statistical Analysis

The normality and homogeneity of data were evaluated by Kolmogorov-Smirnov test and Levene's tests. Analyzed all data with the SPSS program version 23.0 (Chicago, IL, USA). The data analysis method of the growth trial was the independent sample *t*-test, difference was regarded as significant when *P* < 0.05. Meantime, the data analysis method of the muscle cell experimental was one-way ANOVA, determined significant (*P* < 0.05) differences by Tukey's HSD multiple range test. The data of this study was presented as means ± S.E.M. (standard error of the mean).

## 3. Results

### 3.1. Growth Performance

As shown in Table 3, the fish with HL had significantly higher SGR and condition factor (CF) than the fish in the LL group (*P* < 0.05). However, there were no significant differences in survival rate (SR), relative feed intake (RFI), dressout percentage (DP), and body length between LH and LL groups (*P* > 0.05).

### 3.2. Muscle Amino Acid Profiles

Compared with LL group, the content of total amino acids (AA) and nonessential amino acids (NEAA) in fish muscle of HL group had no significant differences (*P* > 0.05), but the content of essential amino acids (EAA) was higher (*P* < 0.05) (Table 4). Among the ten EAA, only methionine (Met), histidine (His), and arginine (Arg) content were not significantly higher in the HL group than in the LL group (*P* > 0.05). Among the NEAA, only the content of glutamate (Glu) in the HL group was significantly higher than that in the LL group (*P* < 0.05), and the rest were not significantly distinct (*P* > 0.05).

### 3.3. Muscle Texture Analysis

From Table 5, we could see the white muscle texture results of fish. The hardness, springiness, chewiness and resilience of the fish fed the HL diet were significantly higher than the fish fed the LL diet (*P* < 0.05). However, the adhesiveness, cohesiveness, and gumminess in the muscle tissue of the fish had no significant changes with the increase of leucine level in the diet (*P* > 0.05).

### 3.4. Histological and Ultrastructure Analysis of Muscle Fibers

**Figure 2:**
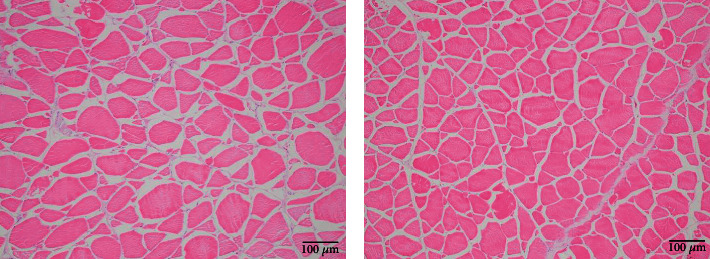
Transversal section of white muscle of blunt snout bream fed the trial diets (means ± SEM, *n* = 4). Photomicrographs (×100) and scale bar (100 *μ*m). The morphology of muscle fiber is irregular polygons. (a) fish fed LL diets; (b) fish fed HL diets.

As shown in Figure 2, the shape of the muscle fibers was an uneven polygon, and the muscle fibers were separated by the endomysium (Figures 2(a) and 2(b)). Combined with Table 6, it can be seen that the muscle fiber density in the HL group was significantly higher than that in the LL group. The percentage of small-sized fibers (<20 *μ*m) in the HL group was significantly higher than that in the LL group (*P* < 0.05).

**Figure 3:**
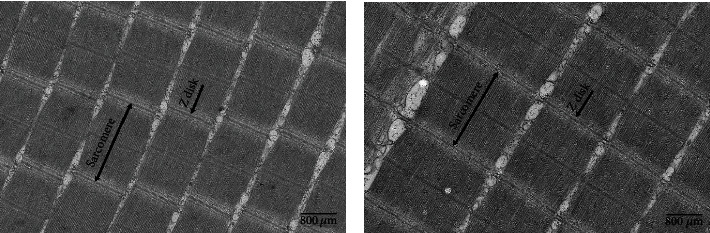
Transmission electron microscope images of white muscle in blunt snout bream fed the trial diets (means ± SEM, *n* = 4). Photomicrographs (×8000) and scale bar (800 nm). The distance between the two-way arrows indicates is the sarcomere lengths (SL). The line pointed by the one-way arrow is the Z disk. (a) fish fed LL diets; (b) fish fed HL diets.

Figure 3 shows the ultrastructure of white muscle myofibrils, the sarcomere length (SL) was the distance between the double arrows indicates (Figures 3(a) and 3(b)). Combined with Table 6, it can be seen that the SL of fish fed the HL diet was significantly longer than that of the fish fed the LL diet (*P* < 0.05).

### 3.5. Gene Expression and Protein Expression in White Muscle

#### 3.5.1. Gene Expression

**Figure 4:**
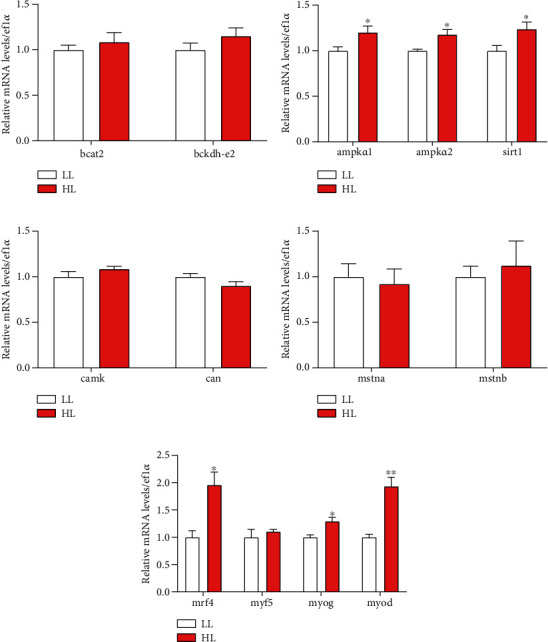
The transcriptional levels of genes involved in energy pathway and muscle fiber development in white muscle of blunt snout bream. Branched-chain amino transferase 2 (*bcat2*) (a); branch-chain *α*-keto acid dehydrogenase E2 (*bckd-e2*) (a); AMP-activated, protein kinase *α*1 (*ampkα1*) (b); AMP-activated protein kinase *α*2 (*ampkα2*) (b); sirtuin 1 (*sirt1*) (b); Calcium/calmodulin-dependent protein kinase (*camk*) (c); Calcineurin (*can*) (c); Myostatin a (*mstna*) (d); Myostatin b (*mstnb*) (d); myogenin (*myog*) (e); myoblast determination protein (*myod*) (e); myogenic regulatory factor 4 (*mrf4*) (e); myogenic factor 5 (*myf5*) (e). Values are represented as mean ± SEM (*n* = 4) by vertical bars. Values marked with asterisks are significantly different (Independent *t*-test, *P* < 0.05).

In Figure 4, no significant differences in branched-chain amino transferase 2 (*bcat2*) and branch-chain *α*-keto acid dehydrogenase e2 (*bckdh-e2*) between the LL group and the HL group (*P* > 0.05) (Figure 4(a)). Dietary HL significantly promoted the expressions of sirtuin 1 (*sirt1*), AMP-activated protein kinase *α*1 (*ampkα1*), and AMP-activated protein kinase *α*2 (*ampkα2*) compared with LL (*P* < 0.05) (Figure 4(b)). The expressions of calcineurin (*can*) and calcium/calmodulin-dependent protein kinase (*camk*) were no significant differences in the groups (*P* > 0.05) (Figure 4(c)). Compared with the LL group, dietary HL had no significant influences on the expressions of (*mstna*), myostatin b (*mstnb*), and myogenic factor 5 (*myf5*) (*P* > 0.05) (Figures 4(d) and 4(e)) compared with LL. With the addition of leucine, the expressions of myogenic regulatory factor 4 (*mrf4*), myoblast determination protein (*myod*), and myogenin (*myog*) in muscle of test fish were improved (*P* < 0.05) (Figure 4(e)).

#### 3.5.2. Protein Expression

**Figure 5:**
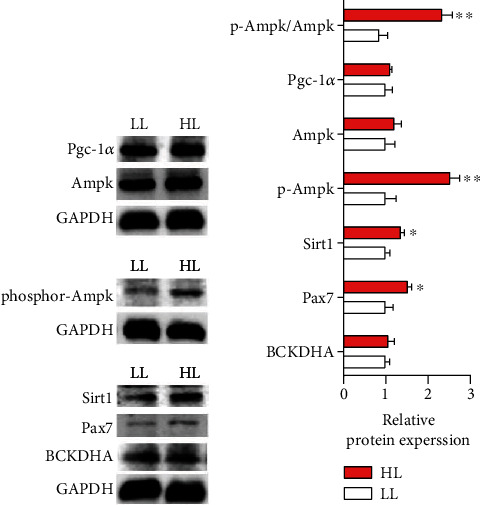
Western blot analysis of BCKDHA, Ampk, p-Ampk, Sirt1, Pgc-1*α*, Pax7, p-Ampk/Ampk in the white muscle of blunt snout bream fed the two kinds of trial diets. Values are represented as mean ± SEM (*n* = 4) by vertical bars. Values marked with asterisks are significantly different (Independent *t*-test, *P* < 0.05).

**Figure 6:**
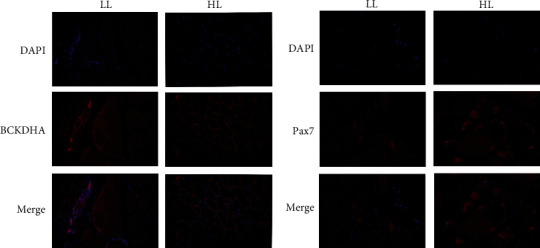
The location of BCKDHA and Pax7 in blunt snout bream white muscle fed the two kinds of trial diets (LL: fish fed LL diets; HL: fish fed HL diets).

The results of muscle protein were displayed in Figure 5. With the addition of leucine, the protein expression of BCKDHA was an insignificant difference (*P* > 0.05) (Figures 5(a) and 5(b) and Figures 6(a) and 6(b)). The protein expressions of p-Ampk, Sirt1 and Pax7 (Figures 5(a) and 5(b) and Figures 6(a) and 6(b)) in the HL group were significantly higher than in the LL group (*P* < 0.05). Meanwhile, the ratio of p-Ampk/Ampk in the HL group was significantly increased (*P* < 0.05). However, the protein expression of Pgc-1*α* showed an insignificant difference in all of the trial groups (*P* > 0.05).

### 3.6. *In Vitro* Results

#### 3.6.1. Proliferation and BCKDH Activity of Muscle Cells

From Figure 1, the viability of muscle cells was the best in the medium with a leucine concentration of 40 mg/L, and the viability of muscle cells diminished with the rise of leucine concentration in the medium after the concentration (*P* < 0.05). The results showed that leucine at a concentration of 40 mg/L could significantly promote the proliferation of muscle cells, but the higher the concentration, the weaker or even inhibited the effect of leucine on muscle cell proliferation (Figure 1).

**Figure 7:**
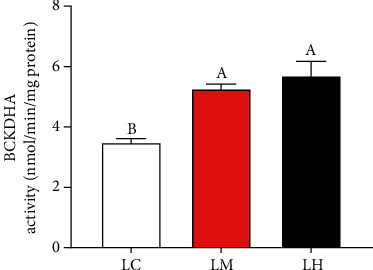
The activity of BCKDH in blunt snout bream muscle cells cultured in the three medium of leucine levels. Values are represented as mean ± SEM (*n* = 4) by vertical bars. Mean values with different letters indicate significantly different (*P* < 0.05).

There was no significant difference in BCKDH activity between LH and LM groups, but both were significantly higher than LC group (*P* > 0.05) (Figure 7).

#### 3.6.2. Gene Expression *In Vitro*

**Figure 8:**
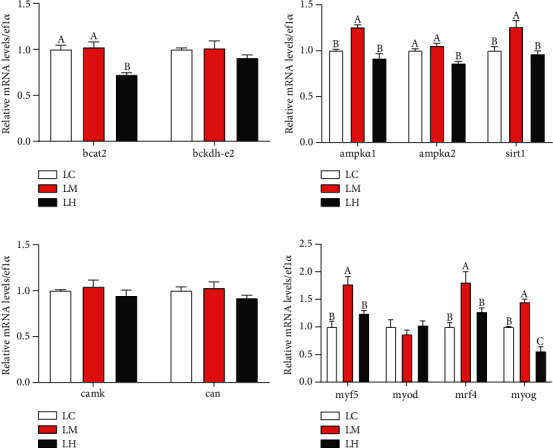
The transcriptional levels of genes involved in energy pathway and muscle fiber development in blunt snout bream muscle cells cultured in the three medium of leucine levels. Branched-chain amino transferase 2 (*bcat2*) (a); branch-chain *α*-keto acid dehydrogenase E2 (*bckd-e2*) (a); AMP-activated, protein kinase *α*1 (*ampkα1*) (b); AMP-activated protein kinase *α*2 (*ampkα2*) (b); sirtuin 1 (*sirt1*) (b); Calcium/calmodulin-dependent protein kinase (*camk*) (c); Calcineurin (*can*) (c); Myostatin a (*mstna*) (d); Myostatin b (*mstnb*) (d); myogenin (*myog*) (e); myoblast determination protein (*myod*) (e); myogenic regulatory factor 4 (*mrf4*) (e); and myogenic factor 5 (*myf5*) (e). Values are represented as mean ± SEM (*n* = 4) by vertical bars. Mean values with different letters indicate significantly different (*P* < 0.05).

The gene expressions of primary muscle cell were shown in Figure 8. The expression of *bcat2* in the LH group was significantly inferior to other groups (*P* < 0.05), but the expression of *bckdh-e2* was no difference among the groups (*P* > 0.05) (Figure 8(a)). The expressions of *ampkα1* and *sirt1* in the LM group were significantly higher than other groups, but the expression of *ampkα2* in the LH group was lower than other groups significantly (*P* < 0.05) (Figure 8(b)). However, the expressions of *camk*, *can* and *myod* were not significantly influenced by the density of leucine (*P* > 0.05) (Figures 8(c) and 8(d)). In addition, *myf5*, *mrf4*, and *myog* expressions in LM group all obtained the highest (*P* < 0.05), and *myog* expression in LH group obtained the lowest (*P* < 0.05) (Figure 8(d)).

#### 3.6.3. Protein Expression *In Vitro*

**Figure 9:**
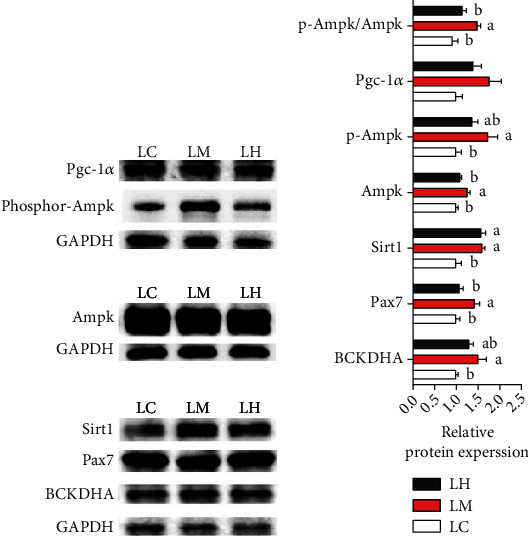
Western blot analysis of BCKDHA, Ampk, p-Ampk, Sirt1, Pgc-1*α*, Pax7, and p-Ampk/Ampk in the muscle cells of blunt snout bream cultured in the three medium of leucine levels. Values are represented as mean ± SEM (*n* = 4) by vertical bars. Mean values with different letters indicate significantly.

As seen in Figure 9, the protein expressions of Ampk and Pax7 were upregulated in the LM group than the groups of LH and LC (*P* < 0.05). The expressions of BCKDHA, p-Ampk in the LM group were significantly higher than LC group (*P* < 0.05). In addition, the ratio of p-Ampk/Ampk in the LM group obtained the highest (*P* < 0.05), and the expression of Sirt1 in the LC group was the lowest. However, the expression of Pgc-1*α* was not different among groups (*P* > 0.05) (Figures 9(a) and 9(b)).

## 4. Discussion

Leucine is an essential amino acid for aquatic animals and plays an important role in their growth and health [19]. At present, it has been reported that increasing dietary leucine level within an appropriate range could improve the growth performance of fish [44–46]. In this study, although the average final weight of experimental fish in the HL group was not significantly higher than that in the LL group (this data is not shown), the SGR in HL group was significantly higher than that in the LL group. It could be seen that moderately increasing the concentration of dietary leucine can improve the growth performance of blunt snout bream. This result was not only the same as Liang's experiment with blunt snout bream [47], but also similar to the above experimental results. It can be seen that the impact of leucine on the growth performance of fish might be slightly similar. In addition, different from the present study, feeding a higher level of leucine (2.15% compared to 1.74%) did not result in further improvement the SGR in juvenile blunt snout bream [23], although had no significant difference. Some discrepancies in the results might be the different initial weight of test fish or different feeding conditions, and the specific reasons require further research. Additionally, CF is an important index to measure body shape, and it is also an index to reflect feed level and life history [48, 49]. Reports on *Channa punctata* [50] and *Bidyanus bidyanus* [51] all pointed out that the CF of fish could be optimized by rising feed nutrient levels. The CF of fish in the HL group was significantly better than that in the LL group. This further demonstrated that the feed with a leucine level of 2.15 has more nutritious for blunt snout bream and was more conducive to the growth of blunt snout bream.

In fish muscle, the mass fraction of protein is about 80% (dry matter), which is the main nutritional component of fish muscle [52]. The composition ratio of amino acids can change the structure and function of proteins [53, 54], thus changing the nutritional value of meat [55, 56]. In the present study, dietary HL had a positive effect on amino acid deposition in fish muscle, especially on EAA. This phenomenon in which dietary leucine levels regulated body deposition of amino acids also occurred in grass carp [13] and juvenile largemouth bass [26]. This indicated that leucine could promote the deposition of essential amino acids in muscle. Additionally, adding more dietary leucine within the requirement of blunt snout bream was more beneficial for the deposition of essential amino acids in the muscle of it in the research. We considered that the difference in the deposition of essential amino acids between the two groups were mainly related to leucine can regulate the turnover of skeletal muscle protein to promote the synthesis of skeletal muscle protein without affecting the degradation of skeletal muscle protein [57].

Muscle fiber characteristics are considered to be the major determinant of fish flesh texture, which contain muscle fiber type, muscle fiber diameter, muscle fiber density, sarcomere length, and so on [58]. Among the characteristics, the muscle fiber diameter and muscle fiber density are crucial determinants of fish flesh texture [59]. Moreover, the texture of fish is an important attribute that reflects the satisfaction of consumers. According to the survey, consumers prefer the firmer flesh texture of fish [60]. In the present study, with increasing dietary leucine level, the density of muscle fiber significantly increased, and hardness also increased. It validated the general rule that muscle hardness is positively correlated with muscle fiber density in fish [61] and indicated that increasing leucine in diet may improve the texture characteristics of blunt snout bream flesh. In addition, it has been reported that muscle hardness and chewiness are inversely correlated with muscle fiber diameter in *Salmo salar* [62] and *Dicentrarchus labrax* L. [2]. In this trial, compared with the LL group, the HL group had a larger proportion of small-diameter fibers, as well as higher muscle texture chewiness and hardness. The above results confirmed that the fish with a high proportion of small-diameter muscle fibers and high muscle fiber density had greater hardness and chewiness, and better flesh quality. In addition, the majority of muscle fiber diameter were less than 20 *μ*m in diameter, suggesting an active proliferative growth process during the developmental stage [63]. Meanwhile, it was indicated that leucine promotes the proliferation of them in mammal muscle cells [64]. Therefore, leucine might regulate fish flesh texture while promoting the proliferation of fish white muscle fibers, thereby optimizing the meat quality of the test fish in the experiment. Furthermore, sarcomeres are the basic contractile units of muscles and are closely connected with flesh water-holding and tenderness capacity. And studies of Wang et al. [65] and Wang et al. [39] have shown that sarcomere length is positively correlated with flesh tenderness. From the results, the sarcomere length of the HL group was increased, and it was also confirmed that high levels of leucine could increase the length of sarcomere to improve the flesh quality of blunt snout bream.

Leucine is a ketogenic amino acid that can oxidize to supply energy. Branched-chain *α*-ketoacid dehydrogenase complex (BCKDH) is the key enzyme in its oxidative metabolism. Increasing the concentration of leucine can activate the activity of BCKDH, start its metabolism, and produce metabolites involved in energy metabolism to supply energy [15]. At the same time, AMP-activated protein kinase (AMPK) is an important energy metabolism sensor in the body of animals, which can be activated by energy, and then activate another energy sensor, sirtuin 1 (Sirt1) located downstream of it [66, 67]. This is called the AMPK/Sirt1 signaling pathway, which can regulate some metabolism processes of the body by regulating the gene expression or activity of downstream molecules [66]. In this study, the mRNA and western expressions of AMPK-related, Sirt1 were greatly raised in muscle cells of the LM group *in vitro*. The results indicated that leucine may activate AMPK and Sirt1 to activate the AMPK signaling pathway. Interestingly, the activity of BCKDH and the protein expression of BCKDHA were increased in the LM group. It indicated that the activation of AMPK and Sirt1 might be related to the metabolism of leucine. *In vivo*, the mRNA and protein expressions of AMPK-related, Sirt1 were greatly raised with the raising of leucine in diet. This also indicated that leucine may activate AMPK and Sirt1 to activate the AMPK signaling pathway. In addition, with the increase of leucine in diet, the expression of BCKDH-related genes and proteins had a slight upward trend, and the transamination product (glutamate) [68, 69] of leucine had increased. These might also imply that the activation of the pathway was related to the metabolism of leucine. As to why the difference was not significant of the expression of BCKDH-related genes and proteins between the LL group and the HL group, might be under a requirement range of blunt snout bream the addition of leucine in the HL group mainly produced the positive influence, and the metabolic environment *in vivo* was more flexible and complex than *in vitro* [70, 71]. However, the specific reasons still need to be further studied. In short, adequate supply of leucine can activity AMPK/Sirt1 pathway which may be related to the metabolism of leucine.

Relative to mammals, increasing the skeletal muscle of fish can coarse existing muscle fibers and add the new [72]. Skeletal muscle regeneration is a complex process, which relies on skeletal muscle satellite cell (SC) and myogenic precursor cells (MPCs), as well as the sequential expression and coordination of multiple factors [73]. Pax7 is specifically expressed in both resting and activated SC, the self-renewal, specialization, and activation of SC in muscle tissue are inseparable from the role of Pax7, and it is also an important marker for identifying SC [74, 75]. Meantime, studies on teleost fish have revealed that the developmental timing and spatial coexpression of myogenic regulators (myogenic regulation factors, MRFs) directly regulate and control the proliferation and enlargement of their muscle cells [4]. MRFs include *myod*, *myf5*, *myog*, *mrf4*, and so on [76]. The *myod* and *myf5* mainly play a considerable role in the determination and maintenance of myoblast, while *myog* and *mrf4* are involved in the differentiation and fusion of advanced myotubes [69]. At the moment, studies with mammals have shown that activation of AMPK can control the expression of genes related to muscle fiber growth, such as *myog* and *mrf4*, to regulate the proliferation and development of muscle fibers [77, 78]. Meanwhile, the Sirt1 can affect the differentiation of muscle fibers by regulating MRFs in mammals [79]. Moreover, Wang et al. [65] and Wang et al. [39] all reported that activation of AMPK/Sirt1 pathway can regulate the expression of some genes of MRFs and thus affect the growth of muscle fibers of *Megalobrama amblycephala*. In this study, LM medium had the best promotion effect on muscle cell viability, and greatly raising the gene expression of *sirt1*, *myf5*, *myog* and *mrf4* and the protein expression of Sirt1 and Pax7 in muscle cells (LM group). At the same time, *in vivo* the gene expression of *sirt1*, *myf5*, *myog* and *mrf4* and the protein expression of Sirt1 and Pax7 were likewise raised by supplementing the dietary leucine. It can be observed that leucine can directly affect isolated muscle cells and regulate the muscle fibers growth and development-related factors of blunt snout bream through the AMPK/Sirt1 pathway. And the reason for the asynchronous expression of *myf5* and *myod in vivo* and *in vitro* might be the overlapping functions of *myod* and *myf5* [80]. Moreover, in *Solea senegalensis* [81], it was indicated that upregulation of *mrf4* was associated with increased early fiber area. In Nile tilapia juveniles [82], it was discovered that *myog* activated muscle proteins and promoted muscle fiber formation. In the present research, *mrf4* and *myog* expression were upregulation in the HL group, which was consistent with the increased muscle fiber density and the percentage of small-sized fibers (<20 *μ*m) in it. The results indicated that providing more leucine within the requirement range of blunt snout bream could promote the continuous replenishment of muscle fibers in it. Meantime, the continuous replenishment of muscle fibers may be related to the activation of AMPK/Sirt1 pathway.

## 5. Conclusion

**Figure 10:**
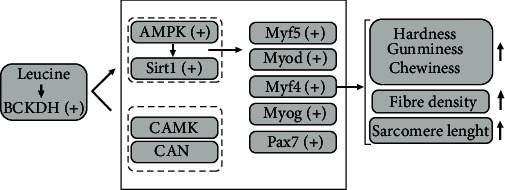
Graphical summary of leucine supplementation increased flesh quality and muscle growth by activating BCKDH and AMPK in blunt snout bream. “+” symbol: expression was upregulated by leucine.

In conclusion, feeding the LH diet increased white muscle hardness, chewiness, springiness and resilience, muscle fiber density, ratio of small fibers (<20 *μ*m), sarcomere length, and expression of genes and proteins related to *in vivo* and *in vitro* myogenesis and muscle growth. It was indicated that leucine supplementation could regulate the growth and development of the muscle fibers of blunt snout bream, thereby improving the flesh quality of it. In addition, leucine supplementation increased BCKDH activity and BCKDHA protein expression *in vitro*, and increased the protein and gene expression of AMPK-related and Sirt1 *in vitro* and *in vivo*. The present results showed that adding more leucine to diet could promote muscle fibers growth and development to improve flesh quality, which may be related to the activation of BCKDH and AMPK (Figure 10).

## Figures and Tables

**Table 1:** Formulation of the different experimental diets (air-dry basis, %).

Diets	LL	HL
*Ingredients (% dry weight*)		
Fish meal	5.00	5.00
Soybean meal	9.00	9.00
Cottonseed meal	15.00	15.00
Rapeseed meal	12.07	12.07
Wheat bran	11.59	11.59
Wheat Middlings	37.00	37.00
Fish oil	2.02	2.02
Soybean oil	2.02	2.02
Premix^a^	1.00	1.00
Ca (H_2_PO_4_)_2_	1.80	1.80
Salt	0.50	0.50
L-leucine	0.00	0.54
Glycine	3.00	2.46
Total L-leucine	1.61	2.15
*Proximate composition (% dry matter)*		
Moisture	10.50	10.60
Crude protein	31.04	32.69
Ether extract	6.49	6.67
Ash	7.97	7.96
L-leucine	1.72	2.39

L-leucine and Glycine purity reached 99% and were purchased from Shandong Gukang Biological Engineering Co., Ltd., Shandong, China. ^a^ Premix supplied the following minerals (g/kg of diet) and vitamins (IU or mg/kg of diet): CuSO4·5H2O, 2.0 g; FeSO4·7H2O, 25 g; ZnSO4·7H2O, 22 g; MnSO4·4H2O, 7 g; Na2SeO3, 0.04 g; KI, 0.026 g; CoCl2·6H2O, 0.1 g; Vitamin A, 900,000 IU; vitamin D, 200,000 IU; vitamin E, 4500 mg; vitamin K3, 220 mg; vitamin B1, 320 mg; vitamin B2, 1090 mg; vitamin B5, 2000 mg; vitamin B6, 500 mg; vitamin B12, 1.6 mg; vitamin C, 5000 mg; pantothenate, 1000 mg; folic acid, 165 mg; and choline, 60,000 mg.

**Table 2:** Nucleotide sequences of primers used to quantify gene expressions by real-time PCR.

Target gene	Forward (5′-3′)	Reverse (5′-3′)	Accession number
*bcat2*	CCAAAGCCAGACCCTTCAAC	GGAAGGGCTTGATCTGAGGT	[83]
*bckd-e2*	AGCCACCATGTGTCGTTTCT	AGTGTCGGGATACGGAGTCA	[83]
*ampkα1*	AGTTGGACGAGAAGGAG	AGGGCATACAAAATCAC	[84]
*ampkα2*	ACAGCCCTAAGGCACGATG	TGGGTCGGGTAGTGTTGAG	[84]
*sirt1*	TCGGTTCATTCAGCAGCACA	ATGATGATCTGCCACAGCGT	[84]
*myod*	TTTGGGCAGCCTCTGGTTC	TGCTTCACCACCCACGACA	[85]
*myf5*	CTGTTGCAGTCAACCATGCT	AGGAACGCCATCCAGTACA	[85]
*myog*	TGGACAGCATTACAGGAACA	TGTTATGGTCGGTGAAAGG	[85]
*mrf4*	GGCAGCCTCTGGTTCGGAT	GCGGAGAGGAGCACGTCCT	[85]
*mstna*	AAGACAACCGGAGACCAAC	ATAGCGTTTCGGAGCAATA	[85]
*mstnb*	GATTTTCGGACTGAAGGAGA	TTCTGGGGGATGACAGTAAG	[85]
*camk*	TGGTGCGGAGATGTGTGAAA	GAGTAGGCGACAGATTCGGG	[39]
*can*	ACCTGGATAACTCGGGCTCT	AAGCTCGCCGTTGGATATGT	[39]
*ef1α*	CTTCTCAGGCTGACTGTGC	CCGCTAGCATTACCCTCC	X77689.1

*bcat2*: branched-chain amino transferase 2; *bckd-e2*: branch-chain *α*-keto acid dehydrogenase E2; *ampkα1*: AMP-activated protein kinase *α*1; *ampkα2*: AMP-activated protein kinase *α*2; *sirt1*: sirtuin 1; *myod*, myoblast determination protein; *myf5*: myogenic factor 5; *myog*: myogenin; *mrf4*: myogenic regulatory factor 4; *mstna*: Myostatin a; *mstnb*: Myostatin b; *camk*: Calcium/calmodulin-dependent protein kinase; *can*: Calcineurin; *ef1α*, elongation factor 1 alpha.

**Table 3:** Survival, growth, feed utilization, and morphometric parameters of blunt snout bream fed the trial diets (Mean ± SEM, *n* = 4).

Indices	LL	HL	*P* value
SR (%)^1^	95.83 ± 2.41	97.92 ± 2.08	0.537
SGR (% day^−1^)^2^	1.57 ± 0.02^b^	1.68 ± 0.04^a^	0.044
RFI (% day^−1^)^3^	2.17 ± 0.03	2.26 ± 0.07	0.328
CF (g/cm^3^)^4^	2.34 ± 0.01^b^	2.57 ± 0.01^a^	*P* ≤ 0.001
Body length (cm)	21.20 ± 0.07	20.88 ± 0.19	0.165

Within the same row, values with different superscripts are significantly, different (Independent *t*-test, *P* < 0.05). ^1^SR (survival rate, %) = 100 × final fish number/initial fish number. ^2^SGR (specific growth rate, %day^−1^) = 100 × [Ln (final body weight) − Ln (initial body weight)]/days. ^3^RFI (relative feed intake, %day^−1^) = feed intake (g) × 100/[(initial fish weight (g) + final fish weight (g)) × 56 days/2]. ^4^CF (condition factor, g/cm^3^) = 100 × body weight (g)/[body length (cm)]^3^.

**Table 4:** Muscle amino acid composition (g/kg of fresh weight) of blunt snout bream fed the trial diets (Mean ± SEM, *n* = 4).

Amino acids	LL	HL	*P* value
*EAA*	9.36 ± 0.13^b^	9.90 ± 0.11^a^	0.019
Leucine	1.51 ± 0.02^b^	1.60 ± 0.02^a^	0.008
Isoleucine	0.83 ± 0.01^b^	0.86 ± 0.01^a^	0.029
Threonine	0.83 ± 0.01^b^	0.87 ± 0.01^a^	0.026
Methionine	0.49 ± 0.02	0.54 ± 0.03	0.226
Valine	0.88 ± 0.01^b^	0.92 ± 0.01^a^	0.018
Tyrosine	0.62 ± 0.01^b^	0.67 ± 0.01^a^	0.020
Phenylalanine	0.76 ± 0.01^b^	0.80 ± 0.01^a^	0.019
Lysine	1.76 ± 0.02^b^	1.87 ± 0.02^a^	0.010
Histidine	0.52 ± 0.01	0.57 ± 0.02	0.113
Arginine	1.16 ± 0.02	1.20 ± 0.01	0.156
*NEAA*	8.30 ± 0.12	8.53 ± 0.11	0.212
Glutamate	2.89 ± 0.04^b^	3.04 ± 0.04^a^	0.031
Glycine	0.95 ± 0.04	0.88 ± 0.02	0.180
Alanine	1.10 ± 0.02	1.13 ± 0.02	0.291
Cystine	0.15 ± 0.01	0.15 ± 0.01	0.864
Serine	0.75 ± 0.01	0.78 ± 0.01	0.255
Aspartic acid	1.87 ± 0.03	1.98 ± 0.03	0.864
Proline	0.58 ± 0.02	0.56 ± 0.01	0.387
*AA*	17.65 ± 0.25	18.42 ± 0.21	0.059

*EAA*: essential amino acids; *NEAA*: nonessential amino acids; *AA*: total amino acids. Within the same row, values with different superscripts are significantly, different (independent *t*-test, *P* < 0.05).

**Table 5:** Texture of white muscle of blunt snout bream fed the trial diets (Mean ± SEM, *n* = 4).

Texture	LL	HL	*P* value
Hardness	645.34 ± 26.40^b^	771.78 ± 30.01^a^	0.010
Adhesiveness	−3.34 ± 0.26	−3.12 ± 0.35	0.640
Springiness	0.43 ± 0.00^b^	0.46 ± 0.01^a^	0.006
Cohesiveness	0.34 ± 0.01	0.35 ± 0.02	0.709
Gumminess	227.98 ± 13.12	230.39 ± 9.09	0.883
Chewiness	104.64 ± 5.81^b^	121.47 ± 3.31^a^	0.031
Resilience	0.12 ± 0.00^b^	0.13 ± 0.00^a^	0.019

Within the same row, values with different superscripts are significantly, different (Independent t-test, *P* <0.05).

**Table 6:** Muscle cellularity and sarcomere length of blunt snout bream fed the trial diets (Mean ± SEM, *n* = 4).

Texture	LL	HL	*P* value
Fiber density (N/mm^2^)	328.03 ± 1.69^b^	338.64 ± 3.02^a^	0.022
% fiber			
<20um	18.00 ± 0.41^b^	19.75 ± 0.25^a^	0.011
20-50um	26.50 ± 0.65	26.00 ± 0.71	0.620
> 50um	55.50 ± 0.65	54.25 ± 0.63	0.215
Sarcomere length (nm)	1,712.97 ± 2.24^b^	1,727.34 ± 2.27^a^	*P* ≤ 0.001

Within the same row, values with different superscripts are significantly, different (Independent *t*-test, *P* < 0.05).

## Data Availability

The data that support the findings of this study are available from the corresponding author upon reasonable request.
